# Two-dimensional echocardiographic measurements of the right coronary artery in healthy horses – a pilot study

**DOI:** 10.1186/s12917-019-1792-1

**Published:** 2019-01-28

**Authors:** Natalia Siwinska, Marcin Michalek, Agnieszka Zak, Malwina Slowikowska, Agnieszka Noszczyk-Nowak, Artur Niedzwiedz, Urszula Paslawska

**Affiliations:** Department of Internal Medicine and Clinic of Diseases of Horses, Dogs and Cats, Faculty of Veterinary Medicine, Wroclaw University of Environmental and Life Sciences, C.K. Norwida 25, 50-375 Wroclaw, Poland

**Keywords:** Equine, Coronary vessel, Arteries, Ultrasound, Heart

## Abstract

**Background:**

Precise understanding of the dimensions of the vascular lumina is essential for accurate interpretation of cardiac vessels imaging. To the authors’ best knowledge, this is the first study focusing on the ultrasound measurement of the right coronary artery (RCA) in the horse. The aim of this study was to determine both the ultrasonographic range of the normal diameter and lumen area of the RCA in horses and the influence of gender, age and level of training on the RCA dimensions. An additional aim of the study was to assess intra- and inter-observer repeatability of the collected measurements.

**Methods:**

Thirty-six privately owned, healthy horses were included in the study. The internal lumen diameter and the area of the RCA were measured in the right parasternal long axis view in the 3rd intercostal space during systole and diastole. The results were compared between groups using the analysis of variance (ANOVA) and Student’s t-test. The correlation between the physiological parameters and the RCA was assessed using Pearson correlation coefficient. Student’s t-test was used to compare the results obtained by two researchers and from two scanners.

**Results:**

The mean diameter of the RCA was 13.1 ± 1.5 mm in systole and 11.5 ± 1.3 mm in diastole, and the mean area was 1.3 ± 0.2 cm^2^ and 1.1 ± 0.2 cm^2^, respectively. There were no statistically significant measurement differences between geldings and mares. A positive correlation between body weight and RCA dimensions as well as height and RCA dimensions were seen. There was a negative correlation between the age and the RCA area. A statistically significant difference in the RCA area was seen between race and retired horses. Intra- and inter-observer agreement was strong with a few statistically significant differences.

**Conclusions:**

The age, size, and level of training may affect the ultrasound measurement of the RCA in horses. Non-invasive transthoracic echocardiography may be used to assess the size of the right coronary vessel in various types of horses.

## Background

The heart needs oxygen and nutrients in order to function properly. These substances are supplied to the cardiac muscle via coronary circulation. The right (RCA) and left coronary artery (LCA) supply different portions of the heart and have varying numbers of branches. Coronary artery dominance is based on these features. Right-dominance has been reported in horses [1, 2]. The dimensions of coronary arteries have biological relevance. However, these structures have received little or no attention in ultrasound examinations in equine cardiology. Precise knowledge of the ultrasonographic size of the vascular lumen is essential to correctly assess the physiology and the anatomy of the vessel in everyday practice. It is also important to know the factors affecting the size of the coronary vessel, which allows for the correct interpretation of the results. Coronary artery anomalies (CAA) may lead to subsequent myocardial ischemia, which is often associated with other underlying diseases [3–13]. While clinically significant disease of the coronary arteries in horses is rare, congenital developmental abnormalities with usually fatal consequences do occur. In addition, understanding coronary artery anatomy and physiology is essential in the study of the cardiac response to exercise and training and of other cardiovascular diseases.

To the authors’ best knowledge, this is the first study focusing on the ultrasonographic RCA measurements in the horse. There have been very few estimates of normal coronary morphology in this species and they have mainly focused on the anatomical structure of the vessel in isolated hearts [1, 2, 14, 15]. There are also numerous studies assessing the effect of age and level of training on the heart size, although none of these focus on equine coronary vessels [16–20].

The aim of this pilot study was to determine the two-dimensional (2D) ultrasonographic diameter and area of the RCA lumen in healthy horses. The four objectives of the study were to determine the influence of (1) sex, (2) age, (3) level of training, (4) and the ratio of heart size to the coronary artery measurements in the horse. Another aim of the study was to determine the intra-and inter-observer repeatability of the obtained results.

## Methods

### Study design

#### Animals

Thirty-six privately owned, healthy horses (18 mares, 18 geldings) aged 4–27 years (mean 12.9 ± 7.6 years old), weighing 336–660 kg (mean 523.3 ± 81.5 kg) with a wither height of 125–176 cm (mean 161.8 ± 10.7 cm) were included in the study. A complete physical examination, non-invasive measurement of systolic blood pressure on the coccygeal artery and non-invasive electro- and echocardiographic examination were performed on each animal. The exclusion criteria were as follows: systemic illness, arterial blood pressure abnormalities, tachycardia or arrhythmia (excluding a physiological second-degree atrioventricular block), audible heart murmurs or any heart abnormality found during echocardiography. The animals were divided into three groups in order to assess the effect of physical activity on the coronary vessel size. Group I contained 10 adult race Thoroughbred horses from four to eight years old (mean 5.5 ± 1.6 years) that regularly participated in flat racing training and races. Group II contained 12 adult animals from 6 to 14 years old (mean 8.9 ± 2.6 years) that regularly participated in two-hour show-jumping and dressage training six times a week and competed at medium level competitions. All the horses in these groups had undergone regular training for at least two years. Group III contained 14 retired horses from 19 to 27 years old (mean 21.6 ± 3.2 years) kept on pastures and previously used only for recreational purposes. The horses had been previously ridden from one to three hours per week but had not trained for at least four years. The II and III group included Warmblood Hanoverian horses, Holsteiner horses, the Wielkopolski horses, and mix-bred horses.

#### Ultrasound examination

**Fig. 1:**
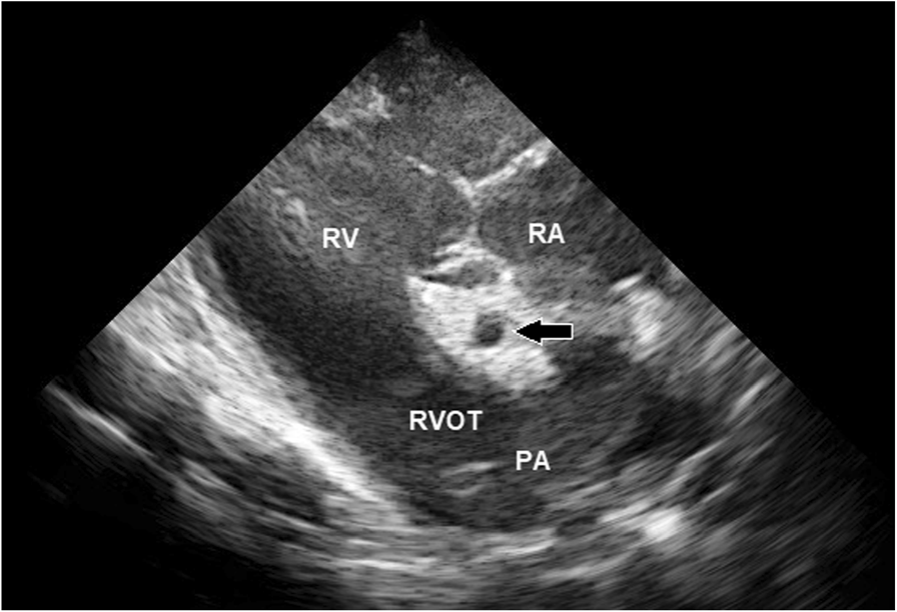
Right parasternal long axis view in 2D ultrasound image of the RCA. The RCA is visible in the middle of the image (white arrow). The right atrium (RA) lies dorsal to the RCA, the right ventricular outflow track (RVOT) is visible lateroventrally to the RCA, the pulmonary artery (PA) is visible ventrally to the RCA while the right ventricle (RV) lies lateral to the RCA

**Fig. 2:**
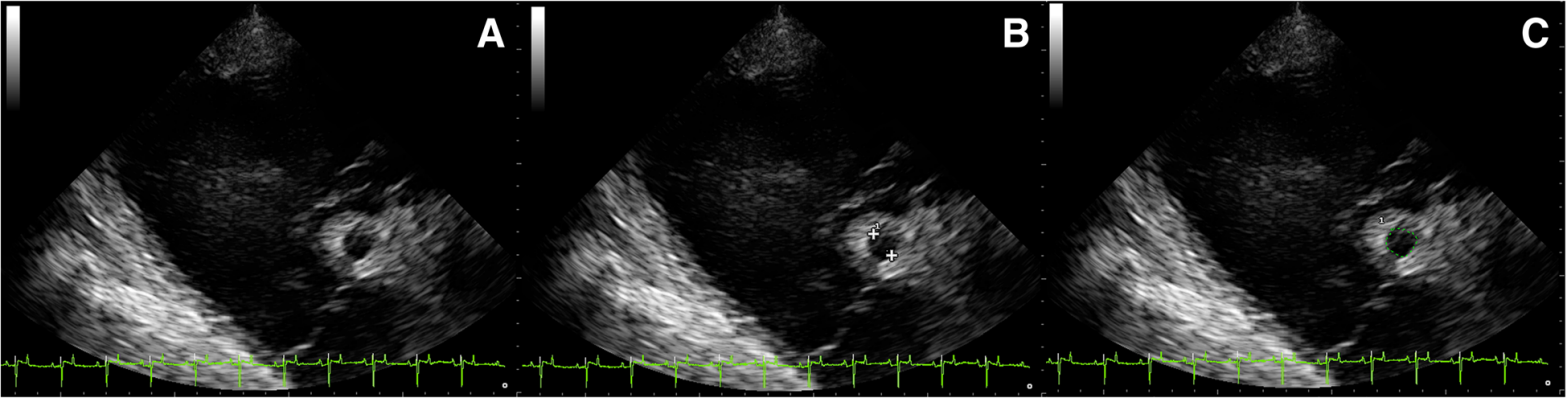
(**a**) 2D ultrasound image portraying the RCA (right parasternal long axis view). (**b**) Imaging of the inner lumen RCA diameter using the inner to inner method. **(c**) Measuring the inner lumen area of the RCA using an ultrasound contour caliper

**Fig. 3:**
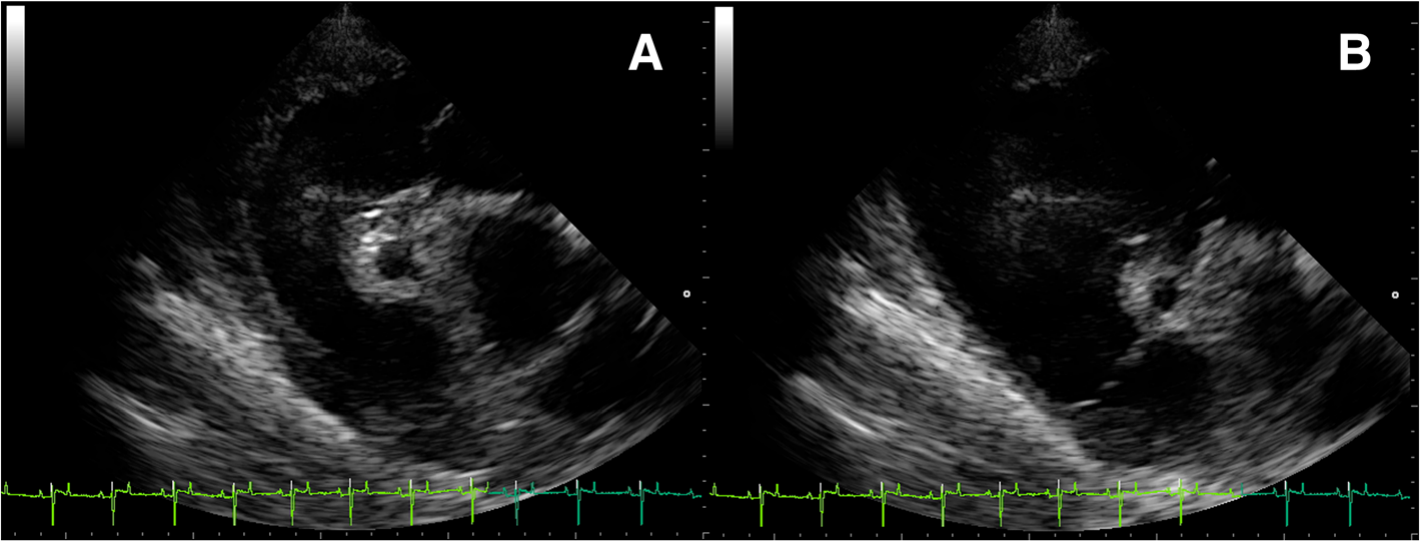
Ultrasound right parasternal long axis view of the RCA in systole **(a)** and diastole **(b)**

Echocardiography was performed using an ultrasound scanner (MyLab™ 30Gold VET, Esoate, Florence, Italy) equipped with a phased array 2.5–5.0 MHz transducer (Esoate, Florence, Italy). A base-apex ECG recording was carried out simultaneously. Prior to echocardiography, the examined site was washed with chlorhexidine-based soap and warm water. Gel was then applied in order to ensure direct contact between the transducer and the skin. Two-dimensional and M-Mode imaging was conducted at an imaging depth of 30 cm from the right hemithorax. M-Mode echocardiograms were obtained from a short-axis view at the LV (left ventricle) chordal level. Three cardiac cycles were measured, and an average value was obtained. The LV internal diameter (LVID), interventricular septal thickness (IVS) and LV free wall thickness (LVFW) were measured using ultrasound calipers during end-diastole (the first ultrasonographic frame, which corresponded to closing of the mitral valve) (d) and end-systole (the first ultrasonographic frame, which corresponded to closing of the aortic valve)(s). The scanner software also calculated the left ventricle mass (LVM), the LV fractional shortening (FS) and the LV ejection fraction (EF). All ultrasound measurements except the RCA measurements were recorded by one researcher (NS). The measurement of the RCA lumen diameter and determination of the internal area by tracing the outline of the lumen were measured in the right parasternal long axis view of the right ventricular outflow track (RVOT) in the 3rd intercostal space following a complete cardiologic examination. The measurements were taken from 2D echo freeze frames visualizing the coronary artery at end-systole and end-diastole using a software-based electronic caliper. The examination was performed uniformly in all the animals. The operator first obtained a cross-section of the aorta. Then, a longitudinal section of RCA was acquired in order to visualize the vessel course, which flowed into the aorta. The operator then proceeded to visualise the transverse plane of the artery at the RVOT level. The RCA was identified based on its appearance and almost central location between the pulmonary artery (PA) and the right atrium (RA) in the examined axis (Fig. 1). After visualising the vessel, the depth of penetration was slightly reduced in order to picture the RCA and to collect all necessary measurements (Fig. 2a). They were taken when an appropriate RCA section was obtained (a circular cross-section of the vessel and the visibility of adjacent structures in a constant cross-section). In horses from the group I, the measurements were collected by a moderately experienced researcher (MS), who was trained to carry out the procedure with the use of the same technique and equipment. Then the main operator (NS) collected measurements of the RCA in group I using the Terason t3000 ultrasound machine (Teratech, Burlington, United States) equipped with a 2.5–5 MHz phased-array transducer. The RCA diameter was measured in millimetres, placing the ultrasound caliper on the inner anterior wall to the inner posterior wall (ITI method; from intima to intima) (Fig. 2b) [21]. The RCA area was measured in square centimetres using the ultrasound caliper to outline the vessel (Fig. 2c). All RCA measurements in systole (Fig. 3a) and diastole (Fig. 3b) were repeated thrice from three different cine loops in each horse. Then, the average was calculated for each horse and for each animal group. All the obtained images were recorded and stored as soft copies (DICOM images). The images were taken at rest and only recorded when the heart rate was consistently ≤40 beats per minute. The study was carried out by licensed researchers. Furthermore, the examinations performed were routine and non-invasive. In accordance with the existing law applicable in Poland, Experiments on Animals Act from January 15th 2015 (Journal of Laws of the Republic of Poland, 2015, item. 266), non-invasive clinical studies do not require ethical approval.

### Statistical analysis

The data are presented as mean ± standard deviation (SD). The one-way analysis of variance (ANOVA) was used to compare age, weight, wither height, heart rate, all heart ultrasound measurements and the RCA diameter and area in systole and diastole between groups. In cases where the hypothesis of equal means was rejected, Fisher’s least significant difference (LSD) *post-hoc* test was carried out in order to determine the difference between the means. In all horses, Student’s t-test for independent samples was carried out to compare the RCA diameter and area between systole and diastole. This test was also used to compare parameters between mares and geldings. Parameter estimation in linear regression analysis and the Pearson correlation coefficient, as well as the subsequent significance, were used to assess the relationship between age, wither height, body weight, HR, heart ultrasound parameters and the mean diameter and internal area of the RCA (in systole and diastole). Inter- and intraobserver repeatability of the RCA diameter and area in systole and diastole was determined based on the Bland-Altman plot. The significance of the differences between the mean results was calculated using Student’s t-test. The coefficient of variation between measurements obtained by two observers as well as by one observer using two ultrasound machines was also calculated. The analyses were performed using Statistica (TIBCO Software Inc., California, USA) for Windows software with a 5% significance level.

## Results

The measurements collected from the studied horses are presented in Table 1.

**Table 1:** Mean values of the clinical examination and measurements of the heart and the right coronary artery using two-dimensional and M-mode ultrasonography in horses divided into three groups: group I – racing horses, group II – sport horses, group III – retired horses

	Group I (*n* = 10)	Group II (*n* = 12)	Group III (*n* = 14)	All (*n* = 36)
Age (years)	5.5 ± 1.6 *^	8.9 ± 2.6 #	21.6 ± 3.2	12.9 ± 7.6
Height (cm)	156.6 ± 3.3	161.9 ± 15.7	163.4 ± 9.2	161.8 ± 10.7
Body weight (kg)	490 ± 61	536.8 ± 98.7	535.4 ± 76.8	523.3 ± 81.5
HR (per min.)	32.8 ± 4.5 *^	37.3 ± 2.9	38.0 ± 3.1	36.3 ± 4.1
IVSd (mm)	32.3 ± 5.1	31.6 ± 7.6	31.2 ± 5.7	31.6 ± 6.1
LVIDd (mm)	114.2 ± 9.5	113 ± 14.4	109.7 ± 14.4	112.2 ± 13
LVFWd (mm)	22.6 ± 2.8 *^	36.5 ± 3.8	26.1 ± 3.4	25.3 ± 3.7
IVSs (mm)	47.5 ± 4.7	46.6 ± 5.9	48.1 ± 7.3	47.5 ± 6.1
LVIDs (mm)	66. ± 7.6	61.2 ± 11	56.4 ± 11.3	60.9 ± 10.9
LVFWs (mm)	43.1 ± 3.6	42.1 ± 7.9	42 ± 5.9	42.4 ± 6
FS (%)	41.9 ± 3.8 ^	45.9 ± 6.8	48.8 ± 5.9	45.9 ± 6.4
EF (%)	77.4 ± 6.4 ^	75.1 ± 7.5	69.4 ± 4.5	74.1 ± 7
LVM (g)	3849.1 ± 483.2	3756.8 ± 1025.5	3601.3 ± 910.5	3721.9 ± 842.3
RCA systole diameter (mm)	13.6 ± 1.3	13.1 ± 1.5	12.8 ± 1.5	13.1 ± 1.5
RCA diastole diameter (mm)	11 ± 1.3	11.8 ± 1.4	11.5 ± 1.3	11.5 ± 1.3
RCA systole area (cm^2^)	1.3 ± 0.2	1.3 ± 0.3	1.4 ± 0.3	1.3 ± 0.2
RCA diastole area (cm^2^)	1 ± 0.2 ^	1.1 ± 0.2	1.2 ± 0.2	1.1 ± 0.2

*statistically significant differences *p* < 0.05 between group I and II; ^ statistically significant differences *p* < 0.05 between group I and III; # statistically significant differences *p* < 0.05 between group II and III

There was a statistically significant difference in age between the groups (*p* < 0.001). The racehorses were significantly younger than the sports horses (*p* = 0.005) and the retired horses (*p* < 0.001), while the sports horses were younger than the retired horses (*p* < 0,001). There was no statistically significant difference in body weight and wither height between groups.

All the studied horses had a sinus heart rhythm and an average of 36.3 ± 4.1 heartbeats per minute. The racehorses had a statistically significantly lower HR (*p* = 0.003) than the sports horses (*p* = 0.017) and the retired horses (*p* = 0.001), as shown in pairwise multiple comparisons. There was a statistically significant difference in the mean LVFWd values between the groups (*p* = 0.022). A pairwise multiple comparisons analysis found a statistically significant difference in this parameter between racehorses and both sports horses (*p* = 0.012) and retired horses (p = 0.017). The FS% and EF% also differed between the groups (*p* = 0.031). Moreover, pairwise multiple comparisons revealed a statistically significant difference in FS% (*p* = 0.009) and EF% (*p* = 0.005) between racehorses and retired horses. There were no other statistically significant differences between the groups in the remaining heart ultrasound parameters.

In all the animals, the cross-section of the RCA was visible in the form of a centrally located circle with an anechogenic lumen surrounded by a hyperechogenic rim. In the right parasternal long axis view, it was located ventrally to the RA, dorsally to the PA and laterally to the right ventricle (RV). There were statistically significant differences between average values obtained during systole and diastole (mean diameter *p* < 0.001; mean area *p* = 0.046). A minimal RCA diameter and area were noted during diastole, while maximal RCA diameter and area values were recorded during systole. The diameter of the RCA in all the animals ranged from 9.3 to 14.5 mm in end-diastole and 11.0 to 16.7 mm in end-systole. The smallest RCA diastolic diameter and the largest RCA systolic diameter were noted in racing horses. The area of the RCA ranged from 0.74 to 1.56 cm^2^ in end-diastole and 0.98 to 1.83 cm^2^ in end-systole. The smallest RCA diastolic area was noted in racing horses and the biggest RCA systolic diameter in retired horses. There were no statistically significant differences in the systolic RCA diameter or the systolic RCA area between young horses in training and the older retired horses. However, there were statistically significant differences between the mean values of the RCA diastolic area (*p* = 0.031), confirmed in multiple comparisons tests (*p* = 0.009).

The relationship between sex, age, wither height, body weight, HR, heart ultrasound parameters, mean diameter and the internal area of the RCA (in systole and diastole) is presented in Table 2.

**Table 2:** The influence of sex on RCA values and the correlation between age, body weight, height, heart dimensions, left ventricle mass and right coronary artery measurements. The correlation coefficient (r) and statistical significance (p)

	Systole				Diastole			
	RCA diameter		RCA area		RCA diameter		RCA area	
Parameter	r	p	r	p	r	p	r	p
Gender	–	0.21	–	0.72	–	0.94	–	0.62
HR	0.248	0.144	0.041	0.814	−0.04	0.819	0.02	0.908
Body weight	0.39*	0.019	0.389*	0.019	0.448*	0.006	0.174	0.31
Height	0.37*	0.026	0.455*	0.005	0.415*	0.012	0.226	0.186
Age	−0.068	0.695	0.317	0.06	0.18	0.294	−0.47*	0.004
IVSd	0.27	0.117	0.34*	0.046	0.23	0.184	−0.02	0.909
LVIDd	0.18	0.301	0.25	0.148	0.14	0.422	−0.12	0.492
LVFWd	0.04	0.82	0.01	0.954	0.22	0.204	0.39*	0.034
IVSs	0.22	0.204	0.28	0.103	0.24	0.165	0.00	0.99
LVIDs	0.18	0.301	0.14	0.422	0.04	0.82	−0.13	0.457
LVFWs	0.2	0.249	−0.01	0.954	0.12	0.492	−0.04	0.82
FS	−0.13	0.457	−0.04	0.82	0.05	0.775	0.1	0.568
EF	−0.14	0.422	−0.05	0.775	0.05	0.775	0.13	0.457
LVM	0.21	0.226	0.21	0.226	0.13	0.457	−0.12	0.492

*correlation present at a *p*-value < 0.05

There were no statistically significant differences between the mean values obtained for mares and geldings. There was a positive correlation between body weight and the RCA diameter in systole and diastole as well as the RCA area in systole. A positive correlation was also observed between the same RCA parameters and height. There was a negative correlation between age and the RCA diastolic diameter. There was no correlation between the RCA parameters and the HR. There was a positive correlation between the diastolic RCA area and the LVFWd as well as between the systolic RCA area and IVSd.

The mean RCA parameters obtained by two different operators and by one operator using two ultrasound scanners are presented in Table 3.

**Table 3:** Intra- and inter-observer measurements of the mean values of the right coronary artery obtained from racehorses (*n* = 10), comparison of the mean values and an analysis of statistically significant differences (*p*-value) as well as the calculation of the coefficient of variation (CV)

	Interobserver					Intraobserver				
RCA	Mean ± SD I	CV (%)	Mean ± SD II	CV (%)	p-value	Mean ± SD I	CV (%)	Mean ± SD II	CV (%)	*p*-value
Diameter systole (mm)	13.65 ± 1.27	9.29	13.7 ± 1.26	9.18	0.688	13.7 ± 1.26	9.18	13.47 ± 1.26	9.38	0.199
Diameter diastole (mm)	10.98 ± 1.27	11.57	11.25 ± 1.37	12.15	0.202	11.25 ± 1.37	12.15	11.48 ± 1.02	8.89	0.579
Area systole (cm^2^)	1.28 ± 0.2	15.23	1.34 ± 0.19	14.18	0.042	1.34 ± 0.19	14.18	1.27 ± 0.18	13.78	0.232
Area diastole (cm^2^)	0.97 ± 0.16	16.79	0.98 ± 0.17	16.99	0.591	0.98 ± 0.17	16.99	1.0 ± 0.12	12.44	0.611

**Fig. 4:**
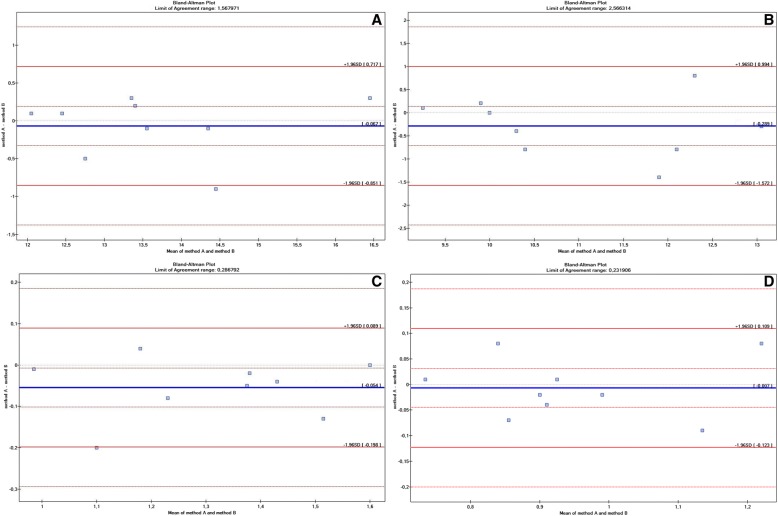
Bland-Altman plots showing the coefficient repeatability of RCA measurements in racehorses obtained by one observer using two ultrasound machines (method A and method B). **a** – RCA diameter in systole; **b** – RCA diameter in diastole; **c** – RCA area in systole; **d** – RCA area in diastole

**Fig. 5:**
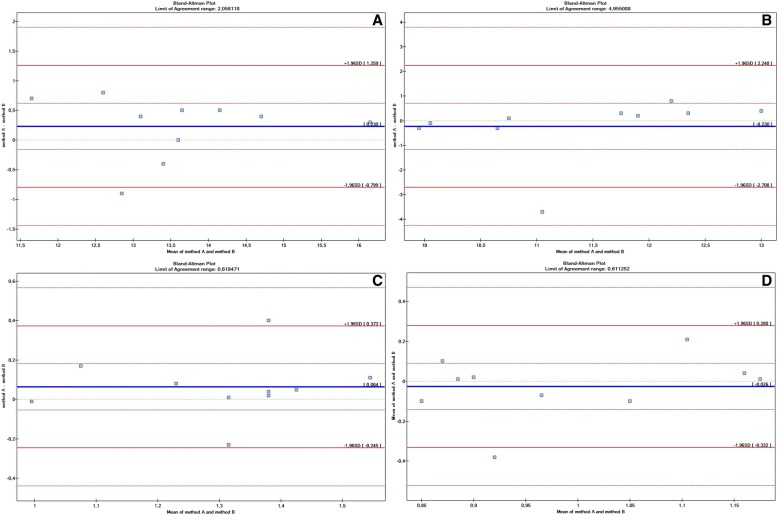
Bland-Altman plots showing the agreement between of RCA measurements in racehorses obtained by two observer using the same ultrasound machines (method A and method B). **a** – RCA diameter in systole; **b** – RCA diameter in diastole; **c** – RCA area in systole; **d** – RCA area in diastole

There were no statistically significant differences in the mean RCA parameters collected by one operator using two different ultrasound devices. However, there were statistically significant differences in the mean RCA area in systole recorded by two operators using the same ultrasound scanner. There were no other statistically significant differences between the results. The Bland-Altman plot showed high repeatability of the RCA measurements obtained by one observer using two ultrasound machines (Fig. 4) and by two observers (Fig. 5).

## Discussion

There is limited data concerning the ultrasonographic characteristics of the RCA in *Equidae*. This is the first study that describes the 2D ultrasonographic measurements of the RCA in healthy adult warmblood horses with different levels of training and of various ages. The RCA, and not the LCA, was used in this study as it is a dominant coronary vessel in the horse and fairly easy to visualize during the ultrasound examination [1, 2]. RCA is a relatively small structure, so it is often difficult to visualize its wall boundaries. Hence, to measure the internal wall diameter, the authors used the ITI method, which, according to Borgjeberg et al. is highly reproducible [21].

The ultrasonographic diameter of the RCA measured in the present study is larger than that of the proximal diameter obtained by Gomez et al. measured in isolated equine hearts (6.72 ± 2.58 mm) [1]. Those authors obtained similar measurements of the LCA (mean diameter 6.76 ± 21 mm) from the same animals [14]. The measurements presented in the current study are also larger than those reported by Thuroff et al. (8.91 ± 0.11 mm) [15]. These differences may be attributed to different methodologies used to measure the vessel (in vitro measurements vs in vivo measurements), the level at which the vessel was measured and the study sample size. In addition, the authors of previous studies carried out measurements of hearts from younger horses up to 3.5 years old and weighing from 250 to 300 kg [1, 14]. The animals from the present study were older and larger. Thuroff observed that coronary arteries demonstrate a somewhat positive allometric growth, indicating that the vessel diameter increases as the animal grows, which is also related to heart growth [15]. The study by Rawlings et al. confirmed this finding as it revealed a positive correlation between the coronary vessel diameter and the size and body weight of the studied ponies [2]. Similarly, Thuroff et al. found a linear correlation between the coronary vessel diameter and that of the virtual heart, associated with the size of the animal [15]. This can be confirmed by the results of the present study, where a positive correlation between the body weight, height and the RCA parameters was found. In humans, there was no correlation between the diameter of the coronary vessels and the body surface area [22]. Differences in the results between horses and humans may be caused by larger weight differences between horses than between humans.

In the presented pilot study, the coronary vessel transverse section size was determined according to the heart cycle. During the ultrasound examination, vessel image movement may be observed depending on the heart cycle, which is related to heart muscle movements and coronary blood flow. There were higher RCA parameters observed during systole and lower ones during diastole. These results reflect coronary flow during a cardiac cycle [23].

Men are reported to have a significantly larger coronary vessel diameter than women [22, 24]. According to that study, men have a statistically significantly larger coronary vessel diameter compared to women. Similarly, Dodge et al. found that the diameter was smaller in women, by on average, 9 + − 8% [24]. There were no statistically significant differences between mare and gelding coronary vessels observed in studies by Gomez et al. on isolated hearts, which is in accordance with the findings of the present study [1, 14]. In the study of Al-haidar et al. (2013), there was no significant effect of gender on any of the echocardiographic dimensional measurements [20]. This may be explained by the fact that there were smaller differences in size between female and male castrated horses of the same breed. Sexual hormones which are present in males (testosterone in particular) might have some influence on the growth and development of several structures. The differences between males and females could have been obtained if stallions had been included in the study. Unfortunately, there were too few stallions available for study during group formation.

In humans and rats, the coronary vessel diameter was found to alter with age due to histological changes in the vessel walls associated with loss of elasticity [25–27]. The most common age-associated vascular remodeling changes include luminal irregularities, an increase in the blood vessel size, lumen dilation, intimal and medial thickening, vascular stiffening or a diameter reduction associated with calcification or hyalinization [25, 28, 29]. In this study, there was a negative correlation between age and the RCA area in diastole, which may be associated with changes similar to those in an old human vessel. However, no statistical differences in the diameter of the RCA lumen were found between older and young horses. These results are in accordance with the findings of Dodge et al. and Fallavollita et al., who also found no statistically significant differences in these measurements based on a population of elderly human patients [24, 29].

It is commonly known that athletic humans and animals have proportionally larger hearts caused by training-induced hypertrophy [16, 24, 30]. Numerous studies have proved differences in cardiac parameters between sports horses and pleasure horses [16–19, 31]. Such hypertrophy is an adaptation of the myocardium to an increased workload induced by intensive, long-term training [16, 30]. Small changes in the endurance component of sports training might result in differences in cardiac measurement within a given training group [17]. Interestingly, despite significant differences in training, age and use of the horses in the present study, there was no statistically significant difference in the mean ultrasound heart measurements between the groups. This might suggest that the training in the studied population of horses was not strenuous enough to cause significant changes in heart dimensions. The mean LVFWd value was the highest in sports horses, while the LVIDs were highest in racehorses, but without any statistical significance. Racehorses also had the highest mean EF% values and the lowest FS% value. This is in accordance with the findings of other authors [17–19, 31]. As the myocardial hypertrophy develops, coronary circulation undergoes a morphological adaptation in order to increase coronary blood flow. The phenomenon of coronary enlargement corresponding to an increase in the left ventricle mass was observed in humans in training [30, 32, 33]. Similarly, Thuroff et al. observed a linear correlation between the diameter of the coronary vessels and the heart mass in animals [15]. Currently, there are no studies on the changes in coronary dimensions in trained healthy horses. In the studied horses, there was no correlation between coronary vessel parameters and ultrasound heart measurements or left ventricular mass. A solitary correlation was noted between LVFWd and IVSd measurements and the RCA area. This coincides with the results of studies conducted in humans, where the thickness of the heart wall was the main determinant for the coronary arteries calibre [30]. In the racehorses in this study, the mean RCA systolic diameter value was the highest while the RCA diastolic diameter was the lowest, although these differences were not statistically significant. The only statistically significant differences between racehorses and retired horses were in the RCA area. The lower average result of this parameter in racehorses compared to non-training horses as well as the highest systolic and the lowest diastolic RCA diameter may result from greater and more efficient vascular and cardiac contractility in this group of animals. Hence, long intensive training may slightly impact the size of the coronary vessels in horses.

Echocardiography is the most widely used imaging method in cardiology and provides information about the morphology and function of almost every cardiac structure. The advancement in ultrasound imaging has improved image quality and enabled 2D visualization of coronary vessels [34]. This method is successfully used to diagnose numerous coronary diseases, mainly due to its availability, non-invasiveness, and relatively low cost. It has been proven that echocardiography is one of the most sensitive methods used to assess the proximal epicardial course of the coronary arteries [30, 34, 35]. Echocardiographic measurement of equine heart size and function show low variability [36]. In the present study, measurements were recorded to assess inter and intra-observer repeatability. The RCA ultrasound measurements were highly repeatable when taken by one operator using two different ultrasound scanners. Similar results were obtained when two operators collected the measurements using the same scanner. However, there was a difference in the RCA area assessment in systole between operators. Carrying out the measurements using the inner wall outline is usually harder and may be subjective. In addition, coronary vessel movements during systole and diastole may affect the captured image and the obtained result. Appropriate skills, training and experience are essential to correctly assess coronary arteries.

Lack of a comparison between the ultrasound results and the dimensions of the vessel collected post mortem is a study limitation. The authors chose to carry out non-invasive transthoracic echocardiography and none of the horses was euthanized. Due to unavailability of transesophageal echocardiography, coronary computed tomography angiography or magnetic resonance imaging, the authors were unable to perform more accurate measurements of the studied structures and compare them with ultrasound results. The second main limitation of the study was the authors’ inability to accurately assess the distance of the measured vessel from the coronary ostium, which may have affected the results. Taking the measurements in field conditions in a timeconstrained environment precluded performing a Doppler ultrasound examination on the RCA, which resulted in recordings only from the right hemithorax. Due to the fact that this was a pilot study, the study groups may have had features of convenient sampling. This may have lead to a degree of sampling error suggesting that the sample may not have been representative of the entire population. In order to further assess equine coronary blood vessels, more recordings should be carried out and they should be combined with other diagnostic techniques. A group of stallions and sick horses should also be examined and the coronary blood flow should be studied using the Doppler technique. There was one more limitation of this study. The measurements were carried out by two sonographers with some experience in collecting similar measurements. The differences in the measurements would have most likely been greater had they been collected by an inexperienced untrained operator.

## Conclusions

These results provide the first RCA ultrasound features in healthy horses. As in humans and other animals, age, weight, height, and level of training can affect ultrasound measurements of coronary vessels in horses. Non-invasive transthoracic echocardiography is a repeatable technique and may be used to assess the size of the right coronary artery in various types of horses if advanced imaging techniques cannot be used. Establishing reference ranges of chosen structures in the ultrasound examination is essential for professionals working with horses.

## Abbreviations

2D: Two-dimensional
CAA: Coronary artery anomalies
d: End-diastole
EF: Ejection fraction
FS: Fractional shortening
ITI: Inner to inner
IVS: Interventricular septal thickness
LCA: Left coronary artery
LV: Left ventricle
LVFW: Left ventricular free wall thickness
LVID: Left ventricular internal diameter
LVM: Left ventricle mass
PA: Pulmonary artery
RA: Right atrium
RCA: Right coronary artery
RV: Right ventricle
RVOT: Right ventricular outflow track
s: End-systole
SD: Standard deviation
